# Pharmacogenomics of Tirzepatide: Genomic Insights into Dual GIP/GLP-1 Agonist Response in Type 2 Diabetes and Atherosclerosis

**DOI:** 10.3390/ph18091261

**Published:** 2025-08-25

**Authors:** Zihang Song, Yifan Tang, Mao Peng, Ruoyu Han, Pingping He

**Affiliations:** 1 Key Laboratory of Model Animals and Stem Cell Biology in Hunan Province, Health Science Center, Hunan Normal University, Changsha 410013, China; 17674905382@163.com (Z.S.); tangyifan0919@163.com (Y.T.); 13787158722@163.com (M.P.); 18113726839@163.com (R.H.); 2 Aging Health Research Center, School of Nursing, Health Science Center, Hunan Normal University, Changsha 410013, China; 3 Engineering Research Center of Reproduction and Translational Medicine of Hunan Province, Hunan Normal University, Changsha 410013, China

**Keywords:** Tirzepatide, pharmacogenomics, type 2 diabetes mellitus, atherosclerosis, genetic polymorphism, precision medicine

## Abstract

Type 2 diabetes mellitus (T2DM) is frequently complicated by atherosclerosis (AS), with substantial overlap in their underlying pathophysiological mechanisms, posing serious threats to patient health. Tirzepatide, a novel dual agonist of glucose-dependent insulinotropic polypeptide (GIP) and glucagon-like peptide-1 (GLP-1) receptors, has demonstrated remarkable efficacy in glycemic control, weight reduction, and cardiometabolic improvement, making it a promising candidate for managing T2DM comorbid with AS. However, substantial interindividual variability in treatment response suggests a role for genetic determinants. This review systematically summarises current evidence on pharmacogenomic variants influencing the efficacy and toxicity of tirzepatide, explores the interplay between drug response genes and genetic susceptibilities to T2DM and AS, and highlights the potential of pharmacogenomics in guiding precision subtyping and individualised therapy. Finally, we highlight key challenges and future directions in the clinical translation of tirzepatide pharmacogenomics, aiming to inform personalized, genomics-guided therapy for cardiometabolic disease.

## 1. Introduction

Type 2 diabetes mellitus (T2DM) is a globally prevalent metabolic disorder characterised by insulin resistance and pancreatic β-cell dysfunction [1]. According to the International Diabetes Federation (IDF) 2021 report, the number of people living with T2DM worldwide has exceeded 530 million and is projected to reach 780 million by 2045 [2]. Importantly, T2DM significantly increases the risk of cardiovascular diseases, particularly atherosclerosis (AS) [3]. AS, one of the most common cardiovascular complications of T2DM, poses a significant threat to both quality of life and life expectancy [4]. T2DM and AS are not only closely linked epidemiologically but also share overlapping pathophysiological mechanisms, including chronic low-grade inflammation, dysregulated lipid metabolism, endothelial dysfunction, and oxidative stress [5]. Evidence indicates that AS progresses more rapidly in patients with T2DM, characterized by more unstable plaques that are prone to triggering adverse cardiovascular events such as myocardial infarction and stroke. This underscores the strong comorbidity and reciprocal aggravation between these two conditions [6].

Although current pharmacotherapies for T2DM and AS—such as insulin, glucagon-like peptide-1 (GLP-1) receptor agonists (GLP-1RAs), sodium-glucose co-transporter 2 inhibitors (SGLT2is), and statins—have diversified in recent years, several clinical challenges remain [7]. Many drugs target isolated pathogenic pathways and often fail to achieve coordinated, multisystemic regulation. Additionally, suboptimal efficacy or treatment-related side effects, such as weight gain, gastrointestinal discomfort, or hypoglycemia, can reduce patient compliance and limit therapeutic success [8,9]. Therefore, the development of multitargeted agents capable of simultaneously managing glycemic control, body weight, and cardiovascular risk represents a pressing need in the era of precision medicine.

Tirzepatide, the first dual agonist of glucose-dependent insulinotropic polypeptide (GIP) and GLP-1 receptors, has demonstrated significant therapeutic potential [10]. In the SURPASS clinical trial program, tirzepatide achieved not only superior glycemic and weight control compared with GLP-1RAs but also a favourable cardiovascular safety profile [11]. Mechanistically, tirzepatide exerts multiple beneficial effects, including stimulation of insulin secretion, inhibition of gastric emptying, appetite suppression, and improvement of insulin sensitivity, rendering it theoretically advantageous for treating patients with coexisting T2DM and AS [12]. However, clinical observations indicate considerable interindividual variability in tirzepatide efficacy and tolerability, suggesting that the genetic background may play a critical role in modulating treatment response [13].

Against this backdrop, pharmacogenomics—an emerging field that explores the genetic basis of interindividual differences in drug response—has gained increasing attention [14]. By identifying genetic variants that influence drug metabolism, transport, and target sensitivity, pharmacogenomics offers key insights into variability in drug efficacy and toxicity [15]. Concurrently, advances in disease genomics have uncovered shared genetic susceptibilities between T2DM and AS, providing a molecular foundation for integrated disease management [16]. The convergence of pharmacogenomics and disease genomics thus presents a novel path toward precision therapy with tirzepatide in the context of T2DM complicated by AS.

This review aims to systematically summarise the current genetic factors associated with tirzepatide efficacy and toxicity, with a particular focus on variations in drug-metabolising enzymes, receptor targets, and signalling pathways. Furthermore, it explores the potential of pharmacogenomic profiling to guide individualised treatment strategies for patients with T2DM and AS comorbidity, offering a theoretical framework to support personalised therapeutic approaches.

## 2. Mechanisms of Action and Clinical Effects of Tirzepatide

**Figure 1:**
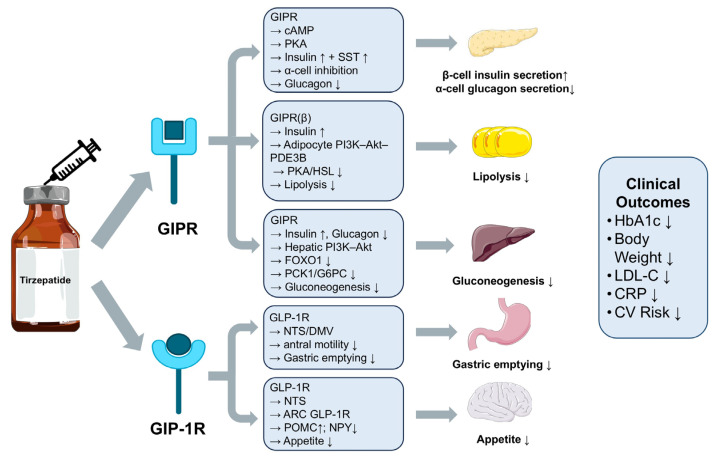
Molecular mechanisms underpinning tirzepatide activity and the corresponding translational clinical outcomes. (DMV, dorsal motor nucleus of the vagus; FOXO1, forkhead box O1; G6PC, glucose-6-phosphatase catalytic subunit; HSL, hormone-sensitive lipase; NPY, neuropeptide Y; NTS, nucleus tractus solitarius; PDE3B, phosphodiesterase-3B; POMC, proopiomelanocortin; SST, somatostatin).

Tirzepatide is a novel dual incretin receptor agonist that exerts synergistic metabolic regulatory effects by simultaneously activating the glucose-dependent insulinotropic polypeptide receptor (GIPR) and the glucagon-like peptide-1 receptor (GLP-1R) [17]. Activation of GIPR enhances pancreatic β-cell function, promotes insulin secretion, and improves insulin sensitivity; meanwhile, GLP-1R activation further inhibits glucagon secretion, delays gastric emptying, and reduces appetite. The combined effects significantly improve glycemic control, weight management, and islet function, overcoming the limitations of conventional GLP-1RAs with single-target activity [10,18] (Figure 1).

The SURPASS clinical trial program (SURPASS-1 to -5) systematically evaluated the efficacy and safety of tirzepatide across different populations with T2DM. In the SURPASS-2 trial, tirzepatide demonstrated superior reductions in HbA1c and body weight compared to the GLP-1RA semaglutide. Notably, the 15 mg dose group achieved a mean HbA1c reduction of up to 2.4% and an average weight loss exceeding 10 kg [19]. SURPASS-4 further confirmed the favourable metabolic effects and cardiovascular safety of tirzepatide in patients at high cardiovascular risk [11]. Other trials also showed that tirzepatide outperformed insulin and other traditional antidiabetic drugs, with good overall tolerability [20]. The main adverse effect was gastrointestinal discomfort, which increased slightly with higher doses [21].

Beyond its glucose-lowering and weight-reducing effects, tirzepatide has been suggested to exhibit potential anti-atherosclerotic properties [22]. Through multiple mechanisms, it may suppress the onset and progression of AS. Firstly, tirzepatide has lipid-lowering effects, reducing total cholesterol, low-density lipoprotein cholesterol (LDL-C), and triglyceride levels, while increasing high-density lipoprotein cholesterol (HDL-C), thereby mitigating lipid deposition in arterial walls [23]. Secondly, it suppresses the expression of pro-inflammatory cytokines such as tumor necrosis factor-alpha (TNF-α) and interleukin-6 (IL-6) and reduces C-reactive protein (CRP) levels, alleviating chronic low-grade inflammation [24]. Moreover, tirzepatide improves insulin resistance and hepatic lipid metabolism, further reducing the metabolic drivers of AS [25]. The combined activation of GIPR and GLP-1R has been observed to attenuate atherosclerotic lesions in transgenic mice expressing apolipoprotein E*3-Leiden (APOE*3-Leiden) and human cholesteryl ester transfer protein (CETP) (APOE*3-Leiden.CETP mice), a humanized dyslipidemic model that develops diet-induced atherosclerosis [26]. This strategy not only improves lipid metabolism and reduces inflammation, but also enhances very low-density lipoprotein (VLDL) clearance efficiency, suggesting its potential application in the prevention and treatment of AS [27].

Importantly, tirzepatide exhibits strong mechanistic complementarity with sodium-glucose cotransporter-2 inhibitors (SGLT2i) [28]. Whereas SGLT2i lowers blood glucose primarily by enhancing urinary glucose excretion and provides established cardiorenal protection, tirzepatide exerts more pronounced effects on weight reduction, insulin sensitivity, and inflammation control. Combining these two agents may potentially yield synergistic benefits in glycemic management, body weight regulation, and cardiovascular and renal outcomes [29]. Preliminary evidence suggests that this combination could potentially improve metabolic parameters and enhance anti-atherosclerotic and anti-inflammatory effects in patients with T2DM and atherosclerosis (AS) [30]. Nevertheless, current clinical data remain limited, and large-scale, long-term studies are warranted to confirm the efficacy, safety, and mechanistic synergy of this therapeutic approach. Overall, tirzepatide, with its unique dual-target mechanism, exhibits broad clinical potential in glycemic control, weight loss, and multifaceted cardiometabolic regulation. It not only provides a more effective therapeutic option for patients with T2DM but also offers new strategies for precision intervention in patients with T2DM complicated by AS.

## 3. Genetic Variants Associated with Tirzepatide Response

Despite the proven efficacy of tirzepatide in glycaemic control and weight reduction, considerable interindividual variability in therapeutic response has been observed [31]. Emerging evidence suggests that this variability is largely attributable to genetic factors, particularly single nucleotide polymorphisms (SNPs) in genes encoding its primary pharmacological targets—GLP1R and GIPR—as well as in genes regulating insulin secretion, adiposity, and appetite control [10]. Polymorphisms in GLP1R and GIPR may affect receptor expression levels, signaling efficiency, and downstream metabolic pathways, thereby influencing drug responsiveness. Table 1 summarizes key genetic variants that have been associated with differential therapeutic responses to tirzepatide. In addition, variants in genes such as Transcription Factor 7 Like 2 (TCF7L2), Fat Mass and Obesity Associated (FTO), and Melanocortin 4 Receptor (MC4R) have been implicated in altered β-cell function and energy homeostasis, further modulating the effects of tirzepatide [32,33]. Understanding these pharmacogenomic determinants is essential for the development of precision medicine approaches, enabling more accurate prediction of treatment response and individualized therapy for patients with T2DM.

### 3.1. GLP1R Genetic Variants and Their Impact on Tirzepatide Response

Tirzepatide partly acts through GLP-1R activation, making GLP-1R gene variants important potential modulators of its efficacy [43]. Among these, the single nucleotide polymorphism rs6923761 (Gly168Ser) is the most extensively studied. This variant has been associated with variable responses to GLP-1RAs. For instance, in T2DM patients treated with gliptins, Ser/Ser homozygotes (A allele carriers) showed significantly smaller HbA1c reductions compared to Gly allele carriers (0.12% vs. 0.80%). Similar findings were reported in GLP-1RA-treated patients, where rs6923761 carriers had attenuated glycaemic responses [43,44]. In obesity treatment, Phan et al. demonstrated that female A allele homozygotes achieved greater weight loss with semaglutide, with evident sex-specific effects [45]. Mechanistic studies suggest that rs6923761 may promote biased agonism, altering downstream receptor signaling and desensitization [46]. Collectively, rs6923761 has been proposed as a promising pharmacogenetic biomarker for predicting tirzepatide response, although direct clinical evidence specific to tirzepatide remains limited and warrants further investigation.

### 3.2. GIPR Genetic Variants and Their Impact on Tirzepatide Response

As tirzepatide partly relies on GIPR activation for insulinotropic and weight-reducing effects, GIPR gene polymorphisms may contribute to variability in its clinical efficacy [37]. In the POUNDS LOST trial, Qi et al. reported that the GIPR variant rs2287019 (C>T) (Effect allele: T) variant influenced metabolic outcomes: T allele carriers showed greater improvements in fasting glucose and insulin resistance following dietary intervention [37]. This suggests that common GIPR variants can modulate GIP-mediated glucose metabolism, potentially affecting tirzepatide response. Supporting this, Saxena et al. identified rs10423928 (T>A) (Effect allele: A) as a GIPR variant associated with altered glucose and insulin responses during oral glucose tolerance test (OGTT) in a large GWAS [38]. Additionally, Jacobi et al. described a rare missense GIPR variant, p.Arg217Leu (rs200485112; Arg→Leu at residue 217), which impaired receptor function and cAMP signaling, highlighting the functional impact of GIPR mutations [47]. Although direct clinical evidence linking GIPR polymorphisms to tirzepatide response is currently lacking, existing studies suggest a mechanistic rationale in which rs2287019, rs10423928, and other GIPR variants could potentially influence tirzepatide efficacy by altering insulin secretion and glucose homeostasis [37,38].

### 3.3. TCF7L2 Genetic Variation and Their Impact on Tirzepatide Response

The TCF7L2 rs7903146 (C>T) variant, a known genetic risk factor for T2DM, has been associated with impaired insulin secretion and glucose metabolism [48]. As tirzepatide promotes insulin secretion through incretin pathways, TCF7L2 variation may influence its therapeutic response. Zeini et al. found that T allele carriers exhibited impaired glucagon suppression during glucose challenge, despite relatively preserved β-cell function [48]. This α-cell dysfunction may reduce tirzepatide’s glycemic efficacy. In addition, Laurenti et al. showed that rs7903146 alters insulin pulsatility, disrupting secretion patterns necessary for effective glucose regulation [49]. Beyond islet function, Verma et al. reported that the T allele impairs adipogenesis via reduced TCF7L2 expression in adipose progenitors, potentially limiting tirzepatide’s weight-loss effects [50]. In conclusion, TCF7L2 rs7903146 is hypothesized to affect tirzepatide response through combined pancreatic and adipose mechanisms, warranting further study.

### 3.4. Obesity-Related Genetic Variants and Their Impact on Tirzepatide-Induced Weight Loss

Tirzepatide’s weight-loss effect is partly mediated through appetite suppression and improvements in lipid metabolism. Variants in key obesity-related genes, such as FTO and MC4R, may influence individual variability in this response [51,52,53]. The FTO rs9939609 A allele is linked to increased food intake and reduced satiety. Dorling et al. reported that carriers have higher ghrelin levels and greater energy intake [51], while Melhorn et al. showed increased reward system activation in response to food cues in these individuals [52]. These observations suggest the possibility that individuals with the FTO A allele may exhibit attenuated anorexigenic responses to tirzepatide. In contrast, MC4R mutations—despite their known role in energy balance—do not appear to diminish the efficacy of GLP-1 receptor agonists. Iepsen et al. found that liraglutide remained effective in promoting weight loss even in patients with MC4R deficiency [53], implying that tirzepatide’s GLP-1R–dependent mechanisms may retain their effectiveness regardless of MC4R status. Thus, while FTO polymorphisms may potentially dampen tirzepatide-induced weight reduction through central appetite dysregulation, MC4R variants appear to be less impactful in this regard.

### 3.5. Genomic Insights from GWAS and Pharmacogenomic Databases

Large-scale genome-wide association studies (GWAS) and curated pharmacogenomics repositories have reinforced the polygenic basis for heterogeneity in GLP-1-based therapeutic response. In a meta-GWAS combining DIRECT, PRIBA, PROMASTER, HARMONY, and AWARD trials, common variants in GLP1R, TCF7L2, and SLC5A2 were significantly associated with glycemic response to GLP-1R agonists [13,54]. These associations support a cumulative small-effect variant model for predicting drug efficacy. Complementary annotations are found in PharmGKB, which flags GLP1R rs6923761 as a pharmacogenetically relevant variant influencing both HbA_1_c reduction and weight outcomes (PharmGKB entry: PA166156820). Recent clinical research further links rs6923761 to sex-specific differences in semaglutide-induced weight loss, underscoring its functional relevance [45]. In addition, Sydney et al. catalog variants in CFTR and SPINK1, recognized as predisposing to drug-related gastrointestinal intolerance or pancreatitis [55]. As tirzepatide enters broader clinical use, integration of GWAS evidence with database annotations will enhance the identification of individuals at risk for suboptimal response or adverse events, paving the way for safer precision prescribing.

### 3.6. Tirzepatide Toxicity and Safety

Tirzepatide is generally well tolerated, but its toxicity profile warrants systematic evaluation. The most common adverse events are gastrointestinal symptoms, including nausea, vomiting, diarrhea, and constipation, which are clearly dose-dependent and represent the main reasons for discontinuation in randomized controlled trials [56,57]. Some patients also experience loss of appetite and rapid weight loss, raising concerns about potential nutritional deficiencies [58]. Similar to other GLP-1 receptor agonists, tirzepatide has been associated with biliary and gallbladder events (e.g., cholelithiasis and cholecystitis), although the overall incidence is low; monitoring is recommended, particularly in individuals undergoing rapid weight loss [19,59]. Pancreatitis, though rare, has been reported in pharmacovigilance databases. Pharmacogenomic studies indicate that certain individuals may be at higher risk: pathogenic CFTR variants (e.g., ΔF508 and R117H) impair pancreatic ductal secretion and increase pancreatitis risk; SPINK1 p.N34S variant weakens trypsin inhibition, conferring an approximately 2.8-fold increased risk of elevated pancreatic enzymes and gastrointestinal symptoms during GLP-1RA therapy [60,61]; moreover, rare variants in PRSS1 and CTRC are associated with earlier onset and progression of chronic pancreatitis. Notably, long-term follow-up data have not shown an increased risk of pancreatic cancer with tirzepatide use [62]. Beyond the pancreas, respiratory safety should also be considered. Although gastrointestinal events remain the primary adverse reactions, some patients may develop respiratory discomfort, such as pneumonia, nasopharyngitis, or influenza [21,63], particularly those with pre-existing chronic lung disease or a history of asthma, warranting close monitoring. Hypoglycemia risk is low with monotherapy but significantly increases when combined with insulin or sulfonylureas, necessitating adjustment of concomitant medications [64]. Additionally, some studies suggest that tirzepatide may be associated with mild increases in heart rate, injection-site reactions, and minor exacerbation of retinopathy, although evidence remains limited [65,66]. Overall, tirzepatide’s toxicity profile is dominated by gastrointestinal adverse events, with biliary and pancreatic events being relatively uncommon [67]. Respiratory events and hypoglycemia mainly occur in patients with pre-existing conditions or concomitant therapies, while individual genetic background may significantly influence pancreatic safety. These findings suggest that risk stratification and long-term monitoring prior to and during treatment may help optimise safety management.

## 4. Genetic Determinants Influencing Tirzepatide Efficacy in T2DM and AS Comorbidity

T2DM and AS frequently co-occur and share common genetic and molecular foundations. Genome-wide association studies have identified overlapping risk loci—including TCF7L2, HNF1A, and CCDC92—that confer susceptibility to both T2DM and AS, highlighting a shared genetic architecture [68]. These variants are involved in pathways regulating glucose and lipid metabolism, endothelial integrity, and inflammatory signaling. Pathophysiologically, chronic hyperglycemia and insulin resistance contribute to endothelial dysfunction, oxidative stress, and macrophage activation, accelerating atherogenesis [69]. Simultaneously, diabetic dyslipidemia, characterized by elevated levels of small dense low-density lipoprotein (sdLDL), promotes foam cell formation and accelerates plaque progression. Inflammatory mediators and advanced glycation end-products (AGEs) further connect the metabolic and vascular dysfunctions underlying both conditions [70]. The convergence of genetic and pathophysiological pathways suggests that susceptibility genes may potentially modulate response to tirzepatide, underscoring the significance of genotype-guided therapy in managing T2DM complicated by AS.

### 4.1. T2DM-Associated Genetic Variants Modulating GLP-1RA Therapeutic Efficacy

Several type 2 diabetes–susceptibility loci modulate the pharmacologic response to GLP-1RAs. Variants in TCF7L2—among the strongest genetic determinants of T2DM—impair β-cell insulin-secretory capacity and thereby influence incretin efficacy; functional studies show that carriers of the rs7903146 risk allele have blunted GLP-1-stimulated insulin release [71], supporting the GWAS findings by Lyssenko et al. [72].

A meta-analysis further demonstrated that susceptibility variants in SLC30A8 and KCNJ11—which affect insulin-granule zinc transport and KATP-channel activity, respectively—alter insulinotropic responses, potentially modifying GLP-1-based therapeutic outcomes [73,74]. These mechanistic insights are supported by independent studies showing reduced first-phase insulin secretion in carriers of the SLC30A8 Arg325Trp variant and impaired GLP-1-stimulated insulin release in KCNJ11 loss-of-function mutants.

Notably, Kyriakidou et al. provided direct pharmacogenetic evidence that the GLP1R coding variant rs367543060, along with additional variants in TCF7L2 and CTRB1/2, predicts variable glycaemic responses to GLP-1RAs among T2DM patients [75]. Collectively, these findings support the hypothesis that T2DM-associated gene variants influence GLP-1RA therapeutic response variability by altering β-cell function and insulin secretion.

### 4.2. AS-Related Genetic Background Shapes Cardiovascular Benefits of Tirzepatide

The individual cardiovascular benefit from GLP-1-based therapies such as tirzepatide may be influenced by genetic variants associated with AS. Among them, APOE gene polymorphisms are well-established contributors to lipid metabolism disorders and residual atherosclerotic cardiovascular disease (ASCVD) risk. Notably, APOE ε4 allele carriers tend to exhibit higher LDL-C levels and vascular inflammation, which may alter therapeutic outcomes [76]. In the SURMOUNT-1 post hoc analysis, tirzepatide administration led to marked improvements in key cardiovascular risk parameters, including reductions in systolic blood pressure, triglycerides, and LDL-C, along with increased HDL-C, thereby lowering the predicted 10-year ASCVD risk [77]. Beyond lipid regulation, tirzepatide also demonstrates vascular-protective effects through modulation of endothelial function, oxidative stress, and inflammation, providing a mechanistic rationale for its benefit in patients with underlying atherosclerotic susceptibility [12]. Furthermore, a recent meta-analysis showed that GLP-1 receptor agonists significantly reduce circulating levels of inflammatory markers such as CRP and IL-6 in T2DM patients, reinforcing their anti-inflammatory and anti-atherosclerotic potential in clinical practice [78].

### 4.3. Disease-Related Downregulation of GLP1R Expression

Pathological states such as diabetes and vascular injury can suppress GLP1R expression, potentially compromising the efficacy of GLP-1 receptor agonists. Kimura et al. demonstrated that GLP1R expression was significantly reduced in the endothelial and smooth muscle cells of db/db mice, with the transcription factor TCF7L2 implicated as a possible upstream regulator [79]. Consistently, a study using a murine arterial injury model revealed that local vascular damage led to downregulation of GLP1R expression in affected vessels, suggesting that mechanical or inflammatory stimuli may alter GLP1R signaling in the vasculature [80]. Moreover, clinical evidence supports the notion that prolonged hyperglycemic states can diminish GLP1R sensitivity, as Kaneto et al. reported that early intervention with GLP-1 receptor agonists may preserve receptor function and confer greater cardiovascular protection compared to delayed treatment initiation [81]. Together, these findings highlight the potential importance of disease context in modulating GLP1R expression and responsiveness.

### 4.4. Toward a Gene–Disease–Drug Interaction Model for Tirzepatide

**Figure 2:**
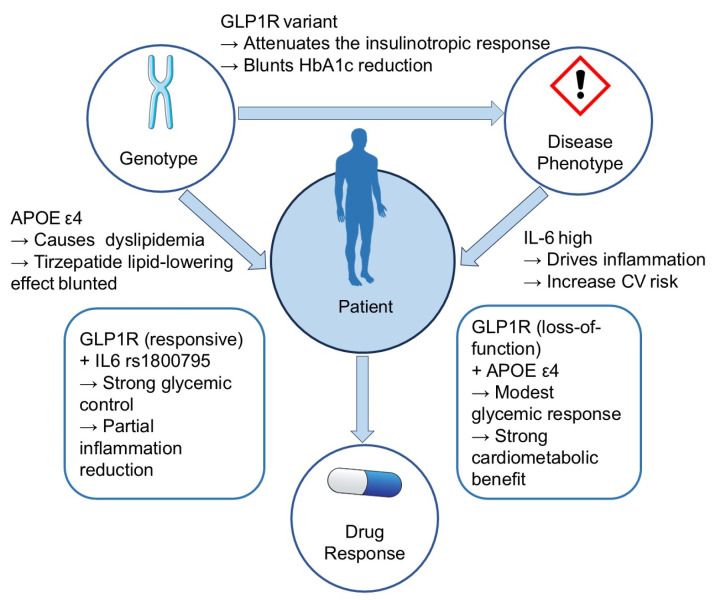
Tirzepatide gene–disease phenotype–drug response interaction network. This multicentric network diagram illustrates the interplay among three core modules—Genotype, Disease Phenotype, and Drug Response. Genotype and Disease Phenotype independently converge on the Patient context, which determines Drug Response and clinical outcomes. Genotype can also shape Disease Phenotype. All arrows represent forward mechanistic influence from upstream to downstream nodes.

Together, these observations support the hypothesis of an integrated gene–disease–drug interaction model (Figure 2). Figure 2 presents a multicentric framework: independent inputs from Genotype (e.g., GLP1R and APOE) and Disease Phenotype (e.g., IL-6–indexed inflammation) converge on the Patient context, which in turn governs Drug Response and clinical outcomes. The arrows represent forward mechanistic influence from upstream to downstream nodes. The single top left-to-right edge indicates that Genotype can also shape Disease Phenotype. The schematic emphasizes direction of influence rather than effect size. Under this framework, an individual’s genomic background (covering both metabolic and vascular-risk loci) intersects with disease phenotype and drug mechanism to shape clinical outcomes. For example, imagine a patient with poorly controlled T2DM who carries a loss-of-function GLP1R variant plus the APOE ε4 allele. The rs6923761 variant has been associated with reduced weight loss and diminished glycemic response to GLP-1 receptor agonists [82]. By contrast, APOE ε4 predisposes to dyslipidaemia and accelerated AS [76].

Tirzepatide’s potent effects on body weight and adipose insulin sensitivity improve LDL-C and triglycerides and also lower high-sensitivity CRP, potentially counterbalancing APOE-driven lipid abnormalities [83]. Moreover, because tirzepatide also activates the GIPR, it can partly bypass GLP-1R insufficiency: GIP signalling still augments post-prandial insulin release and facilitates lipid clearance [84]. As a result, even in the presence of suboptimal glycemic response, patients with an APOE ε4 background may still derive substantial cardiometabolic benefit from tirzepatide.

Conversely, a patient harbouring a highly responsive GLP1R genotype but a pro-inflammatory IL6-174G>C (rs1800795) genotype could achieve excellent HbA1c reduction and weight loss, while only partly suppressing systemic inflammation [85]. In each scenario, the relative “slices” of metabolic versus vascular benefit are dictated by the constellation of risk alleles.

This multilayered view underscores that pharmacogenomic influences extend beyond single variants. Disease-susceptibility genes shape baseline physiology (insulin secretion, lipid handling, and vascular tone), and tirzepatide’s multimodal actions (glucose lowering, weight loss, lipid modulation, and anti-inflammation) intersect with those trajectories. Mapping variant combinations—such as GLP1R + APOE or KCNJ11 + NLRP3—against discrete treatment endpoints will enable finer tailoring of tirzepatide therapy. Ultimately, gene–disease–drug models, supported by integrative resources like GeneDive and other PGx knowledge bases, can drive precision prescribing of dual GIP/GLP-1 agonists, matching patients to the greatest achievable metabolic and cardiovascular advantage [86].

## 5. Pharmacogenomic Stratification and Precision Treatment Potential

Tirzepatide exhibits dual agonism at GIPR and GLP-1R, offering substantial glycemic and cardiometabolic benefits in T2DM complicated by AS. However, inter-individual variability in efficacy and tolerability mandates a stratified, gene-guided approach to optimize outcomes.

### 5.1. Integrating Key Pharmacogenomic Markers

Prospective pharmacogenetic studies underline the impact of GLP1R and GIPR variants on incretin-based therapy response. In a Chinese T2DM cohort treated with GLP-1R agonists, carriers of the GLP1R rs3765467 GG genotype experienced significantly greater HbA1c reduction compared to AA/AG genotypes [87]. Analogously, the GIPR rs10423928 A allele has been associated with enhanced insulin sensitivity and improved glucose homeostasis in subjects with prediabetes [88]. The TCF7L2 rs7903146 T allele, a known T2DM risk variant, alters β-cell incretin sensitivity and impacts the response to GLP-1 receptor agonists [89]. Beyond incretin receptor genes, AS-susceptibility alleles may also affect cardiovascular outcomes. The APOE ε4 allele is linked to dysregulated lipid metabolism and heightened atherosclerotic risk; carriers may derive differing degrees of cardioprotective benefit from tirzepatide’s lipid-lowering and anti-inflammatory effects [90]. Variants in inflammatory cytokine genes such as IL6 may further modulate endothelial function and plaque stability, potentially altering vascular responses to dual agonism [91,92].

### 5.2. Multi-Omics Data Fusion for Enhanced Stratification

Recent advances have accelerated the transition from single-gene pharmacogenomics to integrative, multi-omics frameworks powered by machine learning (ML) for predicting GLP-1RA—and by extension, tirzepatide—therapeutic response. Our framework will integrate genomic variants summarized as polygenic risk scores (PRS), bulk or single-cell transcriptomes, targeted proteomic panels, metabolomic/lipidomic signatures, and clinical/phenotypic features from electronic health record (EHR)/real-world data (RWD); preprocessing will include quality control, normalization with empirical-Bayes batch correction (e.g., ComBat), imputation of missingness using k-nearest neighbors (KNN), or multivariate chained equations as appropriate and feature scaling prior to fusion and modeling [93]. Studies such as Villikudathil et al. demonstrate that combining multi-omic and clinical features with ensemble learners (e.g., random forests) enhances GLP-1RA responsiveness stratification when evaluated by area under the receiver operating characteristic curve (AUROC) and area under the precision–recall curve (AUPRC) [94]. Chen et al. show that autoencoders with latent alignment and attention/transformer modules improve cross-omics representation learning and drug-response prediction (AUC, F1-score) [95]. More generally, attention- and transformer-based multi-omics drug response prediction (DRP) models report gains over baselines [96].

PRS will be computed from GWAS summary statistics using established LD-aware approaches (e.g., LDpred2), standardized within ancestry strata to mitigate portability bias, and entered as features to test incremental value beyond clinical covariates and to enable deployment alongside EHR/RWD [97]. The addition of omic layers refines both mechanistic insight and predictive accuracy: transcriptomic/inflammatory axes involving GLP-1R/GIPR signaling and IL-6 have been implicated in inter-individual incretin biology and GLP-1RA response heterogeneity, and metabolomic/lipidomic profiles—particularly elevated branched-chain amino acids and distinct triacylglycerol species—provide orthogonal markers linked to insulin resistance and cardiovascular risk [98,99]. For high-dimensional feature selection and modeling, we will use sparse, interpretable learners (e.g., LASSO with cross-validated regularization) and hierarchy-aware Priority LASSO to respect modality structure, benchmarked against multiblock and attention-based fusion; performance will be estimated via nested cross-validation with site/center-stratified splits using AUROC, AUPRC, accuracy, sensitivity/specificity for classification (emphasizing PR curves under imbalance), and root mean squared error (RMSE)/mean absolute error (MAE) with R^2^ for continuous endpoints, with calibration (Brier score) and decision curve analysis reported to assess clinical utility (Table 2) [100,101].

Together, these findings underscore the potential of integrated multi-omics and advanced analytics to guide precision prescribing of tirzepatide across heterogeneous T2DM populations.

### 5.3. Personalized Therapeutic Algorithms

Pharmacogenomic stratification enables classification into high, intermediate, and low response groups. For “high responders” (favorable GLP1R/GIPR genotypes, non-risk APOE/IL6 profiles), standard tirzepatide dosing may suffice [102,103]. “Intermediate responders” might benefit from dose escalation or combination with SGLT2 inhibitors to achieve glycemic and cardiovascular targets [104]. “Low responders” or those at risk for gastrointestinal ADRs (e.g., SPINK1 variants) can be pre-emptively managed with anti-emetics or alternative agents, improving adherence and safety [87,88].

In T2DM patients with established AS, integrating pharmacogenomic data can help balance glycaemic control against cardiovascular-risk reduction [105]. For example, an APOE ε4 carrier—whose allele is consistently linked to higher LDL-C and coronary heart disease risk—who also carries the GLP1R rs6923761 A allele, which attenuates GLP-1-mediated glycaemic response, may warrant closer lipid monitoring and earlier adjunctive lipid-lowering therapy when treated with tirzepatide [106,107].

### 5.4. Clinical Translation of Pharmacogenomic Stratification

Translating pharmacogenomic stratification into practice requires validation in large, multi-ethnic cohorts and cost-effective genotyping platforms. The UK Biobank and other biorepositories provide deep genotype–phenotype data to discover and validate marker panels [108]. Developing standardized assays for key SNPs and incorporating results into electronic health records will facilitate point-of-care decision support. In parallel, the development of standardized assays for key variants and the integration of pharmacogenomic results into electronic health records (EHRs) are essential for enabling point-of-care decision support [13].

Practical implementation also depends on clinical interpretability, technical accessibility, and alignment with existing care pathways. Incorporating multi-omics profiles—encompassing variants in GLP1R, GIPR, TCF7L2, APOE, and IL6—has the potential to refine patient selection for tirzepatide and to stratify individuals by both glycaemic and cardiovascular benefit. When embedded within structured clinical workflows, pharmacogenomic stratification has the potential to improve treatment precision, mitigate adverse effects, and support more efficient resource allocation.

## 6. Challenges and Future Directions

Despite the promise of pharmacogenomic stratification for optimizing tirzepatide therapy in T2DM and AS, several critical challenges must be addressed to facilitate its translation into routine clinical practice.

First, the paucity of large-scale, well-powered drug–gene interaction studies limits our understanding of the complex genotype–phenotype relationships underpinning incretin-based therapies. Most GWAS and pharmacogenomic trials disproportionately sample European populations, and large cohorts integrating comprehensive genomic data with detailed drug-response phenotypes remain scarce [109]. Without sufficiently powered, prospective pharmacogenomic investigations, the statistical robustness and clinical utility of candidate SNP panels—including those in GLP1R, GIPR, and inflammation-related genes—cannot be conclusively established.

Second, inter-ethnic variability and drug-response heterogeneity pose significant obstacles. Post-approval analyses of new molecular entities (NMEs) approved by the FDA between 2014 and 2019 revealed that approximately 10% exhibited substantial exposure or response differences across racial or ethnic groups [110]. Allele frequencies of key pharmacogenomic variants (e.g., in CYP2C9 and VKORC1) vary widely among populations and may lead to divergent efficacy or toxicity profiles if not properly accounted for during drug development and labeling.

Third, economic and ethical barriers continue to impede broad clinical adoption. The high costs of comprehensive genetic testing, limited reimbursement mechanisms, and concerns over privacy, informed consent, and potential genetic discrimination have all been identified as key impediments to clinical adoption of pharmacogenomics [111]. Addressing these challenges will likely require coordinated policy efforts to reduce testing costs, enact robust legal protections, and promote patient-centered education to build trust and acceptance.

To overcome these challenges, it is essential to initiate prospective pharmacogenomic trials leveraging large-scale biobanks and longitudinal cohorts. Resources such as the UK Biobank—with deep phenotyping and genome-wide data on 500,000 individuals—provide an ideal platform to correlate genetic variation with drug-response phenotypes in a real-world setting [112]. Comparable initiatives across diverse geographic and ancestral populations (e.g., the All of Us Research Program in the United States and the China Kadoorie Biobank) will be critical to enhancing representativeness and generalizability.

Finally, pharmaceutical companies must integrate gene-stratification strategies early in the drug development process. As highlighted by Manolio et al., the absence of consensus on clinically actionable variants and the lack of standardized genomic knowledge bases continue to hamper the implementation of genomic medicine [113]. Early incorporation of pharmacogenomic endpoints in Phase I/II trials may help identify subpopulations with differential efficacy or safety profiles, informing dose selection, companion diagnostic development, and targeted labeling.

In summary, bridging the gap between pharmacogenomic discovery and clinical application for tirzepatide demands (1) large-scale, multi-ethnic, prospective drug–gene interaction studies; (2) harmonization of global regulatory and reimbursement frameworks; (3) leveraging of biobank infrastructures for comprehensive validation; and (4) proactive adoption of genomic stratification by the pharmaceutical industry. Addressing these areas will be critical to realizing the full potential of precision medicine in T2DM and AS.

## 7. Conclusions

Tirzepatide, the pioneering dual GIP/GLP-1 receptor agonist, offers robust glycemic control and cardiometabolic improvements for patients with T2DM and AS. Genetic variants in key incretin and cardiovascular pathways—such as GLP1R, GIPR, TCF7L2, APOE, and IL6—modulate therapeutic response and safety profiles, highlighting the critical role of pharmacogenomics. Integrating multi-omics data enhances prediction accuracy and could enable precision stratification to optimize efficacy and minimize adverse effects. Future implementation of genomics-guided tirzepatide therapy has the potential to advance personalized medicine, improving clinical outcomes in this complex patient population.

## Figures and Tables

**Table 1:** Key genetic variants influencing therapeutic response to tirzepatide.

Gene	SNP Variant	Associated Effect	Mechanism of Action
GLP1R	rs6923761 (G>A)	↑ delayed gastric emptying	Affecting appetite and postprandial glucose [34]
GIPR	rs1800437 (G>C)	↓ early-phase insulin secretion	Reduces receptor signaling [35,36]
	rs2287019 (C>T)	↓ 30 min insulin	Influence gene–diet interaction [37]
	rs10423928(T>A)	↓ insulin secretion	Modulate glucose metabolism [38]
TCF7L2	rs7903146 (C>T)	↓ insulin secretion	Reduces β-cell responsiveness [39,40]
MC4R	rs17782313 (T>C)	↑ obesity risk	Involved in the regulation of glucose metabolism [41]
FTO	rs9939609 (T>A)	↓ satiety	Affects adipocyte response to satiety signal [42]

**Table 2:** Modalities, preprocessing, algorithms, and evaluation plan for multi-omics pharmacogenomic modeling.

Modality	Example Features	Preprocessing	Feature Engineering/Selection	Fusion/Model Candidates	Primary Metrics
Genomics (PRS)	PRS from GWAS SNPs	Quality control (QC); ancestry-stratified standardization	LD-aware PRS (LDpred2)	Early fusion + LASSO/Priority LASSO	AUROC, AUPRC
Transcriptomics (bulk/scRNA-seq)	Gene counts/pathways	Normalization; batch correction (ComBat)	Variance filtering	Multiblock or attention-based fusion	AUROC, AUPRC
Proteomics	Targeted panel intensities	Normalization; outlier handling	Panel-level z-scores	RF	AUROC, AUPRC
Metabolomics/lipidomics	BCAA; TAG species	Drift correction; scaling	Pathway aggregates	Elastic-net/RF	AUROC, AUPRC
Clinical (EHR/RWD)	HbA1c, BMI, meds, duration	Data harmonization; MICE/KNN	Domain composites (e.g., ASCVD risk)	Multiblock or attention-based fusion	AUROC, AUPRC; calibration (Brier); DCA

## Data Availability

Not applicable.

## Abbreviations

AA, adenine–adenine; ADRs, adverse drug reactions; AG, adenine–guanine; AGEs, advanced glycation end-products; APOE, apolipoprotein E; AS, atherosclerosis; ASCVD, atherosclerotic cardiovascular disease; AUPRC, area under the precision–recall curve; AUROC, area under the receiver operating characteristic curve; CCDC92, coiled-coil domain-containing 92; CETP, cholesteryl ester transfer protein; CFTR, cystic-fibrosis transmembrane conductance regulator; CRP, C-reactive protein; CTRB1, chymotrypsinogen B1; CTRC, chymotrypsin C; CYP2C9, cytochrome P450 2C9; DCA, decision curve analysis; DMV, dorsal motor nucleus of the vagus; DRP, drug response prediction; EHR, electronic health record; FDA, United States Food and Drug Administration; FOXO1, forkhead box O1; FTO, Fat Mass and Obesity-Associated Gene; GIP, glucose-dependent insulinotropic polypeptide; GIPR, glucose-dependent insulinotropic polypeptide receptor; GLP-1, glucagon-like peptide-1; GLP-1R, glucagon-like peptide-1 receptor; G6PC, glucose-6-phosphatase catalytic subunit; GWAS, genome-wide association study; HbA1c, glycated haemoglobin A1c; HDL-C, high-density lipoprotein cholesterol; HNF1A, hepatocyte nuclear factor 1-alpha; HSL, hormone-sensitive lipase; IDF, International Diabetes Federation; KCNJ11, potassium inward-rectifier channel J11; KNN, k-nearest neighbors; LDL-C, low-density lipoprotein cholesterol; MAE, mean absolute error; MC4R, melanocortin-4 receptor; ML, machine learning; NMEs, new molecular entities; NPY, neuropeptide Y; NTS, nucleus tractus solitarius; OGTT, oral glucose-tolerance test; PDE3B, phosphodiesterase-3B; POMC, proopiomelanocortin; PRS, polygenic risk score; PRSS1, serine protease 1; RMSE, root mean squared error; RWD, real-world data; SGLT2, sodium-glucose cotransporter-2; SLC30A8, solute carrier family 30 member 8; SNP, single nucleotide polymorphism; SPINK1, serine-protease inhibitor Kazal type 1; SST, somatostatin; SURMOUNT-1, Study of tirzepatide in participants with obesity or overweight and weight-related comorbidities (Phase 3 SURMOUNT-1 trial); SURPASS, a series of phase 3 studies of tirzepatide in people with type 2 diabetes; TAG, triacylglycerol; T2DM, type 2 diabetes mellitus; TCF7L2, transcription-factor 7-like 2; VKORC1, vitamin-K epoxide-reductase complex subunit 1.
